# Supplementary material for: Multi-Scale Analysis of the Composition, Structure, and Function of Decellularized Extracellular Matrix for Human Skin and Wound Healing Models
Source: Biomolecules. 2022 Jun 16;12(6):837. doi: 10.3390/biom12060837 (PMC9221483; doi:10.3390/biom12060837)
Supplement: Supplementary file 1 [file biomolecules-12-00837-s001.zip › biomolecules-1762794-supplementary.pdf]

**Supporting Information:**

**Table S1. Proteins and Mascot Scores from Mass Spectrometry Data**

| Entry Name | Gene | Name | Mascot Score |
|---|---|---|---|
| A0A287A1S6_PIG | COL1A1 | Collagen Type 1 Alpha 1 chain | 1388.35 |
| F1SFA7_PIG | COL1A2 | Collagen Type 1 Alpha 2 chain | 684.80 |
| A0A286ZQ85_PIG | COL3A1 | Collagen Type 3 Alpha 1 chain | 353.86 |
| A0A286ZVG7_PIG | COL6A3 | Collagen Type 6 Alpha 3 chain | 191.82 |
| A0A286ZVG7_PIG | COL6A3 | Collagen Type 6 Alpha 3 chain | 157.89 |
| A0A5G2Q7R0_PIG | COL6A1 | Collagen Type 6 Alpha 1 chain | 98.17 |
| A0A286ZWS8_PIG | COL2A1 | Collagen Type 2 Alpha 1 chain | 61.06 |
| A0A286ZTA8_PIG | COL5A2 | Collagen Type 5 Alpha 2 chain | 52.32 |
| I3LQ84_PIG | COL6A2 | Collagen Type 6 Alpha 2 chain | 45.93 |
| A0A287BGV6_PIG | COL14A1 | Collagen Type 14 Alpha 1 chain | 37.11 |
| A0A287ART4_PIG | KRT31 | Keratin 31 Type 1 | 448.83 |
| I3LDS3_PIG | KRT10 | Keratin 10 Type 1 | 424.68 |
| F1SGG3_PIG | KRT1 | Keratin 1 Type 1 | 366.01 |
| F1SGG6_PIG | KRT5 | Keratin 5 Type 2 | 324.81 |
| A0A287BH52_PIG | KRT5 | Keratin 5 Type 2 | 271.20 |
| A0A287B5P2_PIG | KRT5 | Keratin 5 Type 2 | 233.56 |
| A0A287AEL2_PIG | KRT14 | Keratin 14 Type 1 | 221.49 |
| A0A5G2QSE8_PIG | KRT3 | Keratin 3 Type 2 | 181.88 |
| A0A287AZL3_PIG | KRT5 | Keratin 5 Type 2 | 179.58 |
| F1S0J8_PIG | KRT14 | Keratin 14 Type 2 | 147.29 |
| A0A287AG48_PIG | KRT7 | Keratin 7 Type 2 | 120.10 |
| A0A287BHY5_PIG | KRT2 | Keratin 2 Type 2 | 118.96 |
| A0A287BEI1_PIG | KRT77 | Keratin 77 Type 2 | 114.17 |
| A0A5G2RBD3_PIG | KRT18 | Keratin 18 Type 1 | 93.95 |
| A0A287APM4_PIG | KRT75 | Keratin 75 Type 2 | 68.28 |
| F1SGI8_PIG | KRT82 | Keratin 82 Type 2 | 34.03 |
| I3LUJ7_PIG | LOC100516036 | Type 2 cuticular Hb6 found in keratin filament (inferred) | 420.38 |
| A0A286ZKZ0_PIG | LOC106507258 | Type 2 cuticular Hb6 found in keratin filament (inferred) | 225.87 |
| I3LK01_PIG | LOC100621639 | Keratin, Type 2 cuticular Hb6-like | 160.59 |
| A0A286ZIH3_PIG | LOC100620900 | Elongation factor 1-alpha, somatic form-like | 64.41 |
| A0A286ZT13_PIG | ALB | Albumin | 232.03 |
| A0A5G2QMD8_PIG | FBN1 | Fibrillin 1 | 138.90 |
| A0A5G2QE62_PIG | VIM | Vimentin | 98.13 |
| I3LL32_PIG | CHIA | Chitinase Acidic | 78.20 |
| A0A286ZRE1_PIG | TPM1 | Tropomyosin 1 | 76.07 |
| A0A287A5G1_PIG | ACTG1 | Actin Gamma 1 | 75.44 |
| A0A286ZN21_PIG | POSTN | Periostin | 74.34 |
| A0A288CFV5_PIG | TF | Transferrin | 74.29 |
| A0A287ASK1_PIG | MYH11 | Myosin Heavy Chain 11 | 53.27 |
| A0A286ZWJ1_PIG | ACTG2 | Actin Gamma 2, Smooth Muscle | 50.56 |
| F1SQ09_PIG | LUM | Lumican | 47.64 |

| | | | |
|---|---|---|---|
| F1SUE4_PIG | ASPN | Asporin | 44.82 |
| F1S6B4_PIG | PRELP | Proline And Arginine Rich End Leucine Rich Repeat Protein | 43.58 |
| A0A0H5ANC0_PIG | OGN | Osteoglycin | 41.65 |
| A0A5G2QUG8_PIG | PSMD1 | Proteasome 26S Subunit, Non-ATPase 1 | 37.68 |
| PEPA_PIG | PGA | Pepsinogen A | 36.08 |
| F1SHX3_PIG | RIF1 | Replication Timing Regulatory Factor 1 | 35.54 |
| I3LBM3_PIG | ZNF329 | Zinc Finger Protein 329 | 35.54 |
| A0A287A3L2_PIG | ECPAS | Ecm29 Proteasome Adaptor And Scaffold | 34.21 |
| A0A287BFI2_PIG | COLEC12 | Collectin Subfamily Member 12 | 33.26 |
| A0A286ZNV3_PIG | SHISAL1 | Shisa Like 1 | 32.92 |
| A0A286ZQC7_PIG | APOA1 | Apolipoprotein A1 | 32.07 |
| A0A286ZV32_PIG | IFT140 | Intraflagellar Transport 140 | 30.95 |
| F1RR71_PIG | GABRA1 | Gamma-Aminobutyric Acid Type A Receptor Subunit Alpha1 | 30.94 |
| A0A287AE06_PIG | LMNA | Lamin A/C | 30.61 |
| A0A286ZT21_PIG | IFIH1 | Interferon Induced With Helicase C Domain 1 | 30.39 |
| APOR_PIG | APOR | Apolipoprotein | 29.49 |
| A0A287ATB8_PIG | WFDC1 | WAP Four-Disulfide Core Domain 1 | 28.70 |
| A0A5G2RDL8_PIG | ATP2C1 | ATPase Secretory Pathway Ca2+ Transporting 1 | 28.70 |
| I3LCJ7_PIG | RPP38 | Ribonuclease P/MRP Subunit P38 | 28.47 |
| A0A286ZUT6_PIG | AKAP9 | A-Kinase Anchoring Protein 9 | 28.23 |
| A0A287AFR2_PIG | GEN1 | GEN1 Holliday Junction 5' Flap Endonuclease | 27.56 |
| A0A287A8C7_PIG | PLEC | Plectin | 27.50 |
| F1SS97_PIG | ANXA9 | Annexin A9 | 27.25 |
| A0A286ZKB1_PIG | CEL | Carboxyl Ester Lipase | 26.47 |
| A0A286ZK41_PIG | IQCE | IQ Motif Containing E | 22.98 |
| A0A286ZRF8_PIG | ANKRD11 | Ankyrin Repeat Domain 11 | 22.53 |
| A0A286ZYB6_PIG | NOS2 | Nitric Oxide Synthase 2 | 21.09 |
| I3LE59_PIG | EME2 | Essential Meiotic Structure-Specific Endonuclease Subunit 2 | 20.39 |

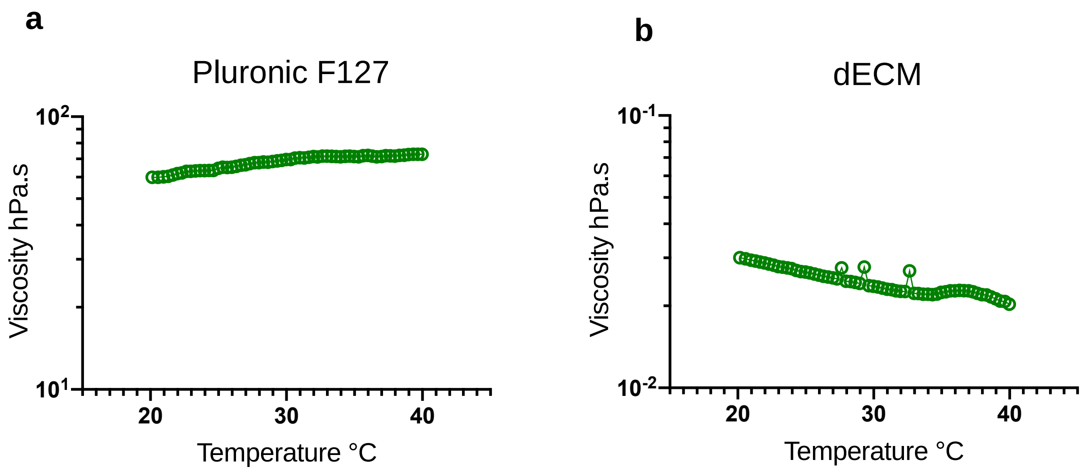


**Figure S1.** Temperature dependent changes in viscosity. Rheological analysis of (A) Pluronic F127 at 25% w/v and (B) dECM.

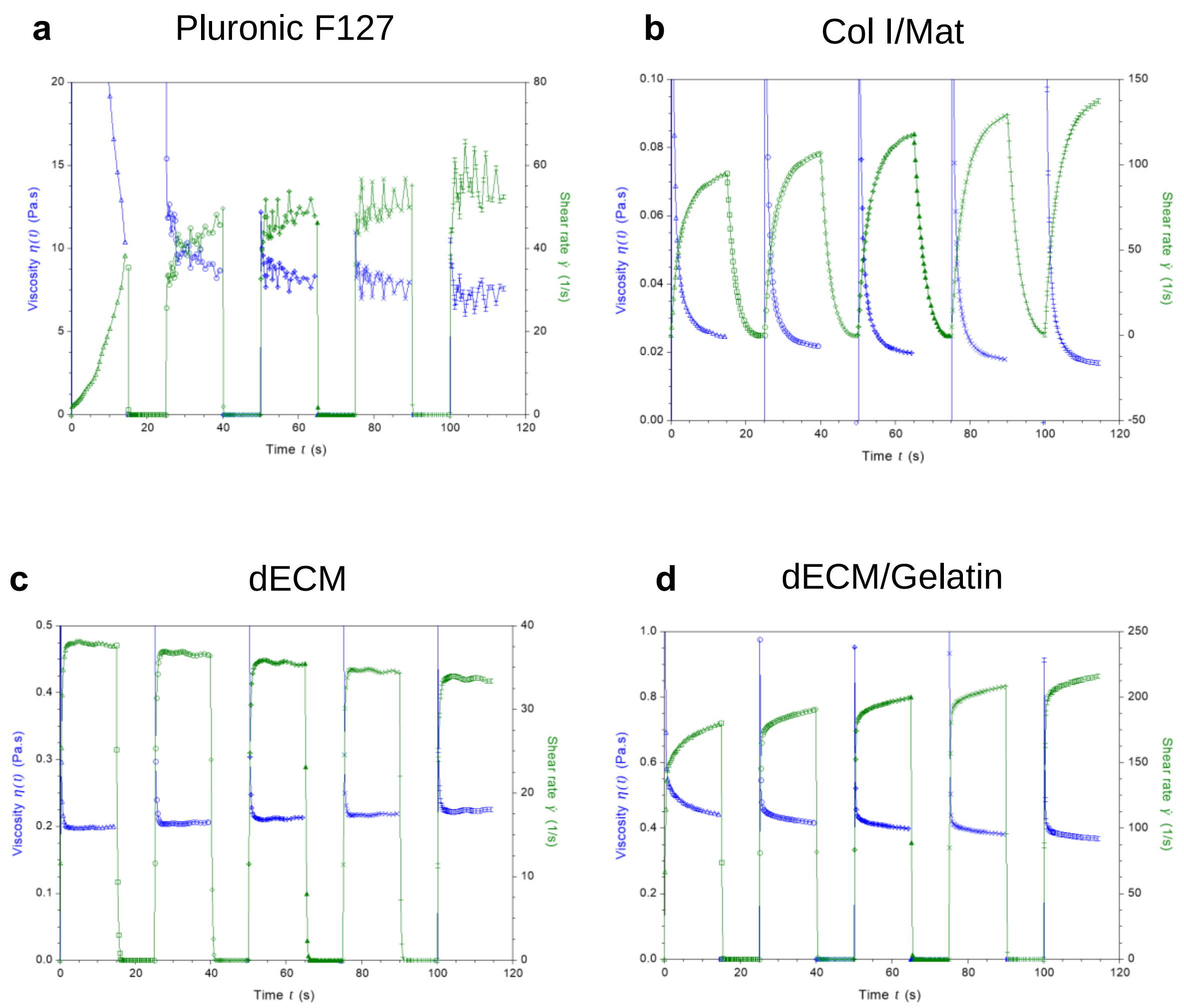


**Figure S2.** Rheological properties of the dECM and dECM/Gelatin hydrogels versus Col I/Mat and 25% Pluronic F127 when subjected to on-off shear rates. Samples were characterized by initially applying a peak hold flow sweep at a shear rate of 50 $s^{-1}$ recording the applied torque. Repeated creep tests were then performed applying the obtained torque for 15 s then releasing for 10 s and repeating this for 20

cycles (these images represent the first 5 cycles of creep and recovery). This data represents two repeat experiments for each material.

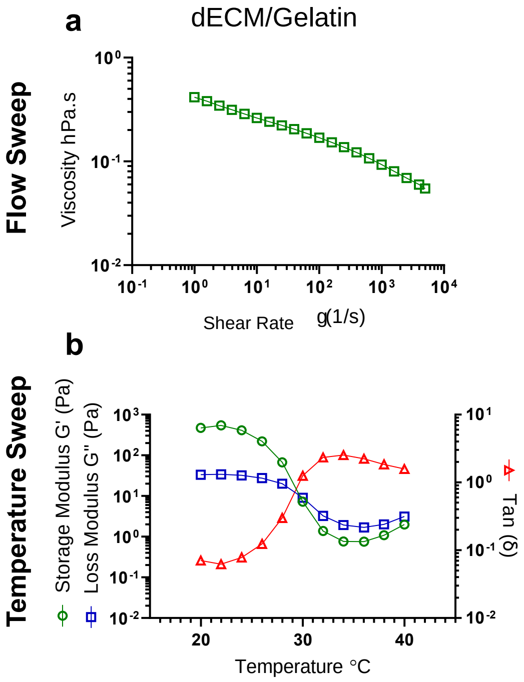


**Figure S3.** Rheological properties of the dECM/Gelatin blend hydrogel. (a) Flow sweep and (b) temperature sweep experiments were performed on a hydrogel bend of 1:1 dECM (20 mg mL$^{-1}$):20% Gelatin. Experiments were carried out on a DHR3 Rheometer (TA Instruments) with the dECM/Gelatin material pre-warmed at 30 °C and neutralized with 1 N NaOH and 10X PBS. Data represents one independent experiment.

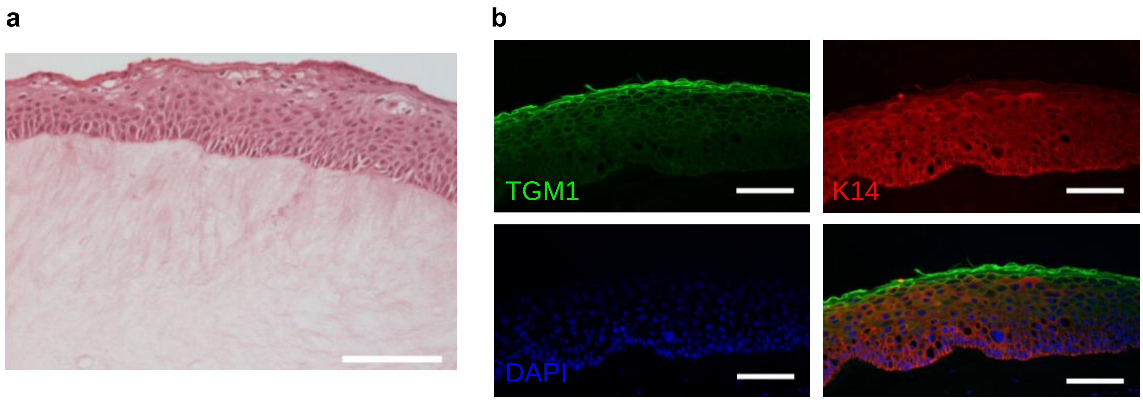


**Figure S4.** Epidermal stratification in HSE models generated in 3D printed frames. (a) H&E image of HSE models constructed using dECM dermal matrix and keratinocytes cultured at the air-liquid interface for 14 days in the 3D printed silicone frames. (b) Representative images of transglutaminase 1 and keratin 14 expression in HSE models. Scale bars equal 100 µm.
